# Protective effect of anti-SUAM antibodies on *Streptococcus uberis* mastitis

**DOI:** 10.1186/s13567-015-0271-3

**Published:** 2015-11-19

**Authors:** Raúl A. Almeida, Oudessa Kerro-Dego, María E. Prado, Susan I. Headrick, Mark J. Lewis, Lydia J. Siebert, Gina M. Pighetti, Stephen P. Oliver

**Affiliations:** Department of Animal Science, The University of Tennessee, Knoxville, TN 37996-4574 USA; Little River Animal and Environmental Unit, East Tennessee AgResearch and Education Center, Walland, TN 37886 USA

## Abstract

In the present study, the effect of anti-recombinant *Streptococcus uberis* adhesion molecule (SUAM) antibodies against *S. uberis* intramammary infections (IMI) was evaluated using a passive protection model. Mammary quarters of healthy cows were infused with *S. uberis* UT888 opsonized with affinity purified anti-rSUAM antibodies or hyperimmune sera. Non-opsonized *S. uberis* UT888 were used as a control. Mammary quarters infused with opsonized *S. uberis* showed mild-to undetectable clinical symptoms of mastitis, lower milk bacterial counts, and less infected mammary quarters as compared to mammary quarters infused with non-opsonized *S. uberis*. These findings suggest that anti-rSUAM antibodies interfered with infection of mammary gland by *S. uberis* which might be through preventing adherence to and internalization into mammary gland cells, thus facilitating clearance of *S. uberis*, reducing colonization, and causing less IMI.

## Introduction

Environmental streptococci, particularly *Streptococcus uberis*, account for a significant proportion of mastitis in lactating and nonlactating cows [1–3], and heifers [4]. Current prevention and control programs originally designed for the control of contagious mastitis pathogens such as *Streptococcus agalactiae* are only marginally effective against *S. uberis*. Susceptibility to *S. uberis* mastitis varies during the different stages of the lactation cycle, showing the highest prevalence during the early nonlactating and periparturient periods [5, 6].

Research conducted in our lab lead to the discovery of a novel *S. uberis* virulence factor identified as *S. uberis* adhesion molecule (SUAM) [7]. SUAM is a fibrillar surface protein associated with the *S. uberis* cell wall by a hydrophobic region, and has affinity for lactoferrin (LF). Further in vitro studies showed that SUAM plays a central role during the early events of *S. uberis* IMI via adherence to and internalization into bovine mammary epithelial cells (BMEC). Mechanisms underlying the pathogenic involvement of SUAM rely partially on its affinity for LF, which together with a putative receptor on the surface of BMEC creates a molecular bridge which facilitates adherence to and internalization of *S. uberis* into BMEC [7–9]. We also discovered that SUAM has a LF-independent domain that also mediates adherence and internalization, and that anti-SUAM antibodies blocked both pathogenic mechanisms [9]. Further studies using a SUAM deletion mutant showed that adherence and internalization of the SUAM mutant strain into BMEC was markedly reduced as compared with the parent *S. uberis* strain [10].

In an attempt to enhance mammary immunity during the late nonlactating and periparturient periods, we conducted a vaccination study using recombinant SUAM (rSUAM) as antigen. Results showed that significant increases in anti-rSUAM antibodies in serum and mammary secretions can be achieved during these high mastitis prevalence periods [11]. Furthermore, vaccination-induced anti-rSUAM antibodies inhibited in vitro adherence to and internalization of *S. uberis* into BMEC [11]. The purpose of the present study was to extend our observations by using an in vivo approach to evaluate the effect of anti-rSUAM antibodies on the pathogenesis of *S. uberis* IMI.

## Materials and methods

### Antibody production

Recombinant SUAM was purified as described [11]. Concentrated rSUAM was sent to Quality Bioresources, Inc. (Seguin, TX, USA) for production of antibodies. Anti-rSUAM antibodies were affinity purified from sera of rSUAM-immunized steers using rSUAM conjugated to Ultra Link Biosupport (Thermo Scientific, Rockford, IL, USA) and eluted with 0.1 M citrate buffer. Final antibody concentration as determined by ELISA was 21.0 mg/mL.

### Bacterial strain, culture conditions and preparation of challenge suspension

*Streptococcus uberis* UT888, a strain originally isolated from a cow with chronic mastitis, was used in this study [1]. Frozen stocks of *S. uberis* UT888 were thawed in a 37 °C water bath, streaked onto blood agar plates (BAP), and incubated for 16 h at 37 °C in a CO_2_: air balanced incubator. A single colony from the BAP culture was used to inoculate 50 mL of Todd Hewitt broth (THB, Becton–Dickinson, Franklin Lakes, NJ, USA) and incubated for 16 h at 37 °C in an orbital rocking incubator at 150 rpm. The resulting suspension was then diluted in PBS (pH 7.4) to a concentration of 4.0 log_10_ colony forming units/mL (CFU/mL), mixed with anti-rSUAM antibodies at a final concentration of 15.0 mg/mL and further incubated for 1 h at 37 °C. The challenge suspension used for positive control mammary quarters was prepared in parallel but omitting the addition of anti-rSUAM antibodies.

### Challenge protocol

Twenty mastitis-free (negative bacteriological culture and milk SCC <250 000 cells/mL at quarter level) Holstein cows in their 2nd and 3rd lactations and in their first 60 days of the lactation were used. Cows were allocated randomly to the experimental (*n* = 10) or positive control (*n* = 10) groups. One mammary quarter of each cow in the experimental group was infused with *S. uberis* UT888 opsonized with affinity-purified anti-rSUAM antibodies (opsonized *S. uberis*). One uninfected mammary quarter of cows in the control group was infused with non-opsonized *S. uberis* UT888. Non-infused quarters were used as negative controls. The experimental IMI protocol was approved by The University of Tennessee Institutional Animal Care and Use Committee.

### Clinical assessment of animals following challenge

Challenged cows were monitored twice daily during the 1st week (CH0 through CH + 7), and once daily at CH + 10 and CH + 14. During these inspections, rectal temperature, clinical assessment of milk and mammary glands, as well as local signs of inflammation were monitored and recorded. Milk and mammary scores were evaluated using a scoring system described in Table 1.

**Table 1:** Mammary gland and milk evaluation and scoring.

Score	Milk appearance	Mammary score			Demeanor
		Palpation	Temp.	Color	
0	Normal	Pliable, light	Normal	Normal	None
1	Flakes	Slight, firmness, swelling	Normal	Normal	None
2	Clots	Firm, moderate swelling	Warm	Red	None
3	Stringy, watery, bloody	Hard, severe swelling	Hot	Red	Uncomfortable, irritable, kicks

Mammary quarters were considered infected and classified as IMI as described [12]. Subclinical mastitis was defined as quarters without clinical signs having positive isolation of *S. uberis* (≥500 colony forming units per mL (CFU/mL)) and/or corresponding increase of SCC (>2.5 × 10^5^). Clinical mastitis was defined as quarters having scores of >2 for milk and mammary appearance.

### Milk sample evaluation

Samples of foremilk were collected aseptically from each mammary quarter 7 days before challenge (CH − 7), immediately before challenge, twice daily at milking from CH0 through CH + 7 and once daily at CH + 10 and CH + 14. Microbiological evaluation of milk samples was done following procedures recommended by NMC. Identification of *S. uberis* strains used was as described [4, 13]. Milk somatic cell counts (SCC) were analyzed at the Dairy Herd Improvement Association Laboratory, Knoxville, TN, USA.

### Statistical analysis

Data on mammary scores, SCC and bacterial counts were analyzed using SAS software (Cary, NC, USA). A mixed model repeated measures (autoregressive variance structure) with cow as the subject was used to compare strains, time, and their interaction.

Least squares means were separated using Fisher’s protected LSD at the 5% significance level. Variables were examined for normality (Shapiro–Wilk >0.90) and equal variance, which showed bacterial counts needed log transformation.

## Results

### Mammary scores

**Figure 1:**
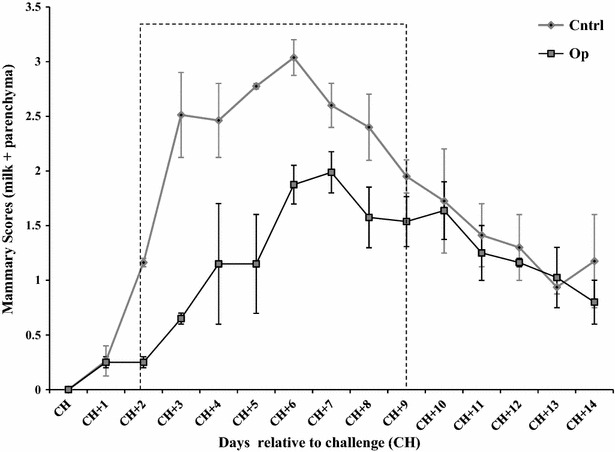
**Milk and mammary parenchyma scores of challenged quarters.** Twenty uninfected bovine mammary quarters were challenged with *S. uberis* UT888 opsonized with anti- rSUAM antibodies () or the untreated *S. uberis* UT888 () and milk and mammary scores were obtained. Data are the sum of milk and mammary scores and each data point represents the mean of two daily observations for all challenged quarters in each group. Error bars are the standard error of the mean (SEM). Values included in the dotted line box were statistically significant (*P* ≤ 0.05).

Inflammatory changes in milk and mammary quarters infused with opsonized *S. uberis* were significantly lower than in cows infused with non-opsonized *S. uberis* (positive control group) (Figure 1). Mammary quarters infused with non-opsonized *S. uberis* began to show clinical signs of mastitis 36 h post-challenge, reaching the highest milk appearance and mammary score/demeanor at 3 (CH + 3) and 6 (CH + 6) days post-challenge, respectively. In contrast, mammary quarters infused with opsonized *S. uberis* had the highest milk appearance and mammary score 1 week post-challenge. During days 2–9 post-challenge, mammary scores of quarters infused with opsonized *S. uberis* were significantly lower than changes observed in mammary quarters infused with non-opsonized *S. uberis* (*P* ≤ 0.05). No scores were detected in milk or mammary gland parenchyma of non-infused quarters (negative controls).

### Microbiological findings

**Figure 2:**
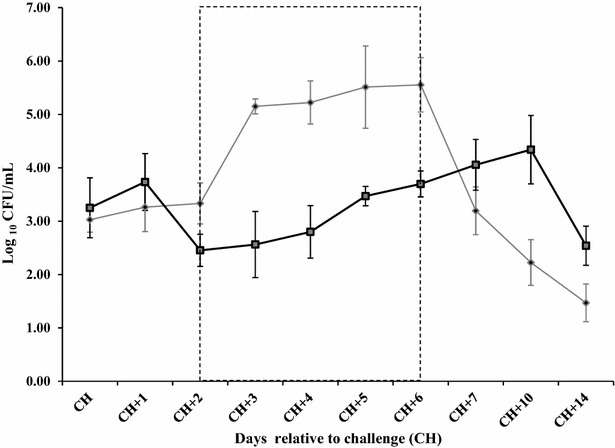
**Log**_**10**_ **colony forming units/mL (CFU/mL) of** ***S. uberis*** **UT888.** Twenty uninfected bovine mammary quarters were challenged with *S. uberis* UT888 opsonized with anti-rSUAM antibodies () or untreated *S. uberis* UT888 () and CFU/mL in milk of challenged quarters were obtained. Data are presented as log_10_ CFU/mL and are the mean of daily observations for the 1st week and at 10 and 14 days post-challenge for each treatment group. Error bars are the standard error of the mean (SEM). The dotted line box includes time points where differences between groups were statistically significant (*P* ≤ 0.05).

Milk from mammary quarters infused with opsonized *S. uberis* had significantly lower bacterial counts than quarters infused with non-opsonized *S. uberis* (Figure 2). In mammary quarters challenged with non-opsonized *S. uberis,* the maximum numbers of bacteria in milk were detected on days 3 and 6 post-challenge and were about 2.5 log_10_ higher than values used to challenge mammary quarters. In contrast, during the same period (CH + 2–CH + 6), numbers of bacteria in milk from mammary quarters infused with opsonized *S. uberis* were significantly lower than the corresponding number for the control group (*P* ≤ 0.05). No bacteria were isolated from milk of the negative controls quarters.

### Somatic cell counts

**Figure 3:**
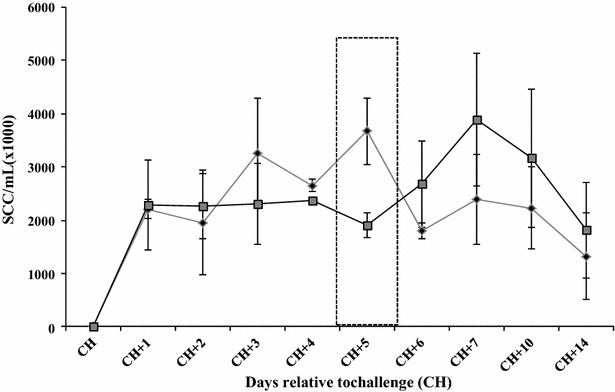
**Somatic cell counts (SCC) in milk from challenged quarters.** Twenty uninfected bovine mammary quarters were challenged with *S. uberis* UT888 opsonized with anti-rSUAM antibodies () or untreated *S. uberis* UT888 () and SCC/mL in milk of challenged quarters were obtained. Data are daily values obtained during the first 7 days following challenge (CH) and error bars are the standard error of the mean (SEM). The dotted line box includes differences statistically significant (*P* ≤ 0.05).

Somatic cells counts in milk of challenged quarters of the opsonized and control group, increased markedly after challenge and continued increase throughout the observation period (Figure 3). Somatic cell counts of the opsonized group were lower than these of the control groups reaching statistically significant level at CH + 6 and CH + 7 (*P* ≤ 0.05).

### Clinical signs

**Figure 4:**
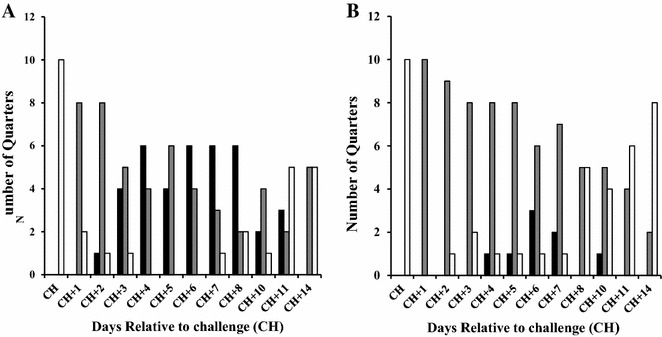
**Intramammary infections (IMI) detected in mammary quarters infused with non-opsonized** ***S. uberis*** **UT888 or opsonized with anti-rSUAM antibodies.** Infused mammary quarters were infused with non-opsonized *S. uberis* UT888 (**A**) or opsonized with anti-rSUAM antibodies (**B**) and monitored daily for 2 weeks. Mammary quarters were classified as clinical () subclinical () or negative () based on milk appearance and mammary scores, and microbiological data. Bars indicate the number of IMI detected in each group.

Sixty percent of mammary quarters infused with non-opsonized *S. uberis* developed clinical mastitis and such percentage was significantly higher as compared to mammary quarters infused with opsonized *S. uberis* (Figure 4). Intramammary infections (IMI) were detected in all quarters infused with the non-opsonized strain and in 10% of the quarters infused with *S. uberis* treated with anti-rSUAM antibodies. In addition, by the day 14 after challenge, IMI was detected in 50% of the quarters. In contrast, 20% of the quarters of the opsonized group had IMI. Only mild clinical signs of mastitis were observed in mammary quarters infused with opsonized *S.* uberis and while 50% of the control cows required antibiotic therapy, no treatment was needed for cows in the opsonized group (data not shown).

## Discussion

In a previous communication, we reported a novel virulence factor from *S. uberis* identified as *S. uberis* adhesion molecule (SUAM) [7]. Further research showed that this molecule had a central role on adherence and internalization of *S. uberis* into BMEC and that anti SUAM antibodies from immunized cows were able to reduce adherence to and internalization of *S. uberis* into BMEC [11]. Even though these results were very promising, the lack of data generated from in vivo approaches was the piece missing in our research. To solve this void, we conducted an in vivo passive protection assay to specifically answer the question about the protective effect of anti-SUAM antibodies.

Passive immunity is the transfer of antibodies from one individual to another and occurs naturally when maternal antibodies are transferred to the fetus through the placenta, or when antibodies specific for a pathogen or toxin are passively transferred to achieve immediate protection against a specific pathogen [14]. Passive protection is the status obtained by passive immunity and assays directed to test the efficacy of specific antibodies to neutralize pathogens or toxins are known as in vivo passive protection assay. Typically, in vivo passive protection assay consists of treatment of susceptible individuals with specific antibodies before experimental exposure to the target pathogen. Protective effect of the test antibodies is determined by measuring the reduction of symptoms or progression of the disease as compared to non-treated controls [15–17]. In this study, we used a variation of such a method. In our approach, *S. uberis* was opsonized with anti-rSUAM antibodies prior to infusion into healthy mammary glands of dairy cows and similarly as a control the same non-treated strain infused into healthy mammary glands. Results showed that mammary quarters infused with *S. uberis* opsonized with anti-rSUAM antibodies had less clinical mastitis, with mild symptoms, and lower bacterial counts in milk as compared to control quarters. Somatic cells counts and bacterial counts in CFU/mL were lower in mammary glands infused with *S. uberis* opsonized with anti-rSUAM antibodies from CH + 2 to CH + 5. In spite of these differences, by CH + 10 CFU/mL were higher in milk of quarters infused with opsonized *S. uberis* that in the control group. Such differences could be due to the fact that in absence of active production of anti-SUAM antibodies, a fraction of *S. uberis* not affected by the blocking effect of these antibodies or innate defenses of the mammary gland follow the pathogenic pathways of *S. uberis* IMI, resulting in augmented CFU/mL in the milk of these cows. It is important to note that the concentration of anti-rSUAM antibodies used (15.0 mg/mL) was about 5 times more concentrated than normal IgG values (~3 mg/mL) during the peripartum period in dairy cows, as reported [18]. This suggests that optimization of local antibody responses through strategic vaccination schedules and routes of administration need to be achieved in order to confer effective protection during the peripartum period.

Findings reported in this communication indicate that anti-rSUAM antibodies have a protective effect against *S. uberis* IMI, possibly either by blocking adherence and internalization of *S. uberis* into host cells [11], and/or likely by mediating the *S. uberis* phagocytosis by neutrophils and macrophages in the mammary glands. These findings confirm our previous in vitro observations about the protective role of anti-rSUAM antibodies [11] and establish the value of our in vitro experimental model based on cocultures of BMEC with *S. uberis* as an initial step in identification of *S. uberis* virulence factors.

In conclusion, results from this investigation demonstrated that anti-rSUAM antibodies partially protected mammary glands from *S. uberis* infection following experimental challenge most likely by preventing adhesion and invasion of bacteria into host cells and/or through opsono-phagocytic removal of bacteria by phagocytic cells.
